# Capacity-Building Through Digital Approaches: Evaluating the Feasibility and Effectiveness of eLearning to Introduce Subcutaneous DMPA Self-Injection in Senegal and Uganda

**DOI:** 10.9745/GHSP-D-24-00019

**Published:** 2024-10-29

**Authors:** Siri Wood, Ericka Roberts, Aurora Anna Stout, Alain Kaboré, Allen Namagembe, Jane Cover, Marème Dia Ndiaye, Mouminatou Diokh, Farmata Sèye, Beth Balderston

**Affiliations:** a PATH, Seattle, WA, USA.; b PATH, Dakar, Senegal.; c PATH, Kampala, Uganda.; d Formerly of the Ministry of Health and Social Action, Dakar, Senegal.

## Abstract

Training health workers is one of the biggest challenges and cost drivers when introducing a new contraceptive method or service delivery innovation. PATH developed a digital training curriculum for family planning providers who are learning to offer subcutaneous DMPA (DMPA-SC), including through self-injection, as an option among a range of contraceptive methods. The DMPA-SC eLearning course for health workers includes 10 lessons with an emphasis on informed choice counseling and training clients to self-inject. In partnership with Ministries of Health in Senegal and Uganda, the course was rolled out in select areas in 2019–2020, including during the COVID-19 pandemic when physical distancing requirements restricted in-person training. We conducted evaluations in both countries to assess the practical application of this digital training approach for contraceptive introduction. The evaluation consisted of a post-training survey, an observational assessment conducted during post-training supportive supervision, and an estimation of training costs.

In both countries, a majority (88.6% in Uganda and 64.3% in Senegal) scored above 80% on a DMPA-SC knowledge test following the training. In Senegal, where there was a comparison group of providers trained in person, those providers scored similar on the post-test to eLearners. Providers in both groups and in both countries felt more prepared to administer DMPA-SC or offer self-injection to clients after receiving a supervision visit (93%–98% of eLearners felt very prepared after supervision as compared to 45%–72% prior). The evaluation results suggest that digital approaches offer a number of benefits, can be cost-effective, and are most optimal when blended with in-person training and/or supportive supervision.

## Abstract (precis)

This evaluation of online training for health workers to counsel clients wishing to self-inject subcutaneous DMPA suggests that online training can be effective while saving time and money. Further, eLearning courses work best when complemented with supportive supervision to help health workers correctly apply their knowledge through hands-on practice.

## Teaser

**Key Findings:**

- This evaluation found that eLearning can be an effective training approach for diverse health care provider cadres, including community health workers and pharmacists, as demonstrated by high scores on the subcutaneous DMPA knowledge assessment and good injection proficiency (measured by health care worker’s implementation of the 4 critical steps).
- Post-training supervision for eLearning trainees was found to be crucial. This aligns with the World Health Organization’s recommendations for digital training that complements, rather than replaces, traditional in-service training for health systems strengthening.

**Key Implications:**

- The results of this evaluation highlight that to extend our digital learning capacities, more investment should be made to develop the necessary support systems for eLearning to reach health workers with limited or no access to smartphones or computers, who live in areas with weak network connectivity, and/or who have lower literacy levels.
- Program managers should consider using eLearning approaches in combination with in-person skills-building sessions and on-site supervision to optimize mastery of skills by learners.

## INTRODUCTION

Training health workers is one of the biggest challenges and cost drivers when introducing a new contraceptive method or service delivery innovation, typically representing 50% or more of program costs.^1^^–^^3^ Up to half of those training costs go toward meals and accommodation when building health workers’ capacity through traditional classroom-based trainings.^4^ However, eLearning—a method that uses modular online content to support self-directed and self-paced learning on a smartphone, laptop, desktop, or other mobile device—presents potential as an effective and cost-efficient approach to training health workers.^5^^–^^7^

The COVID-19 pandemic presented new challenges, as physical distancing requirements made implementing traditional, in-person training difficult. Although the Internet has allowed people to connect remotely for some time, the realities of the pandemic have prompted renewed focus on eLearning as a viable alternative to in-person training by harnessing digital platforms to advance the introduction and scale-up of new contraceptive methods and service innovations. Findings from eLearning evaluations in low- and middle-income countries (LMICs) in recent years concluded that for eLearning to be effective, it should be accessible, effective, convenient, cost-efficient, interactive, and tailored.^7^^–^^11^ This may be particularly true in LMICs and rural settings where geographical distance, inadequate staffing, resource limitations, and costs can impede training access.^1^^,^^7^^,^^12^ Additionally, eLearning helps reduce continuity of care challenges that occur when providers leave health care facilities for multiday trainings.^13^ However, creating the optimal environment for successful eLearning can be challenging in LMICs that may lack stable Internet connectivity, universal access to mobile technology, digital literacy, or funding for the potentially high costs of mobile phone or Internet data plans required to implement eLearning as the sole training approach.^3^^,^^7^^,^^14^

This article describes findings from a multicountry evaluation of an eLearning program for the introduction of a relatively new version of injectable contraception, subcutaneous DMPA (DMPA-SC). DMPA-SC is a lower-dose, easy-to-use injectable contraceptive that protects against pregnancy for 3 months and can be administered by a provider—including those with a low level of training—or self-injected by trained clients. In 2020, PATH rolled out an eLearning course for health care providers in English and French in partnership with ministries of health in Uganda and Senegal, 2 countries that are working to scale up access to DMPA-SC, including self-injection (SI), by increasing the number of service delivery points actively offering the contraceptive option. PATH also conducted evaluations in both countries to assess the practical application of using this digital approach to train providers on DMPA-SC. The evaluations aimed to understand whether the eLearning approach was effective, feasible, and cost-efficient; for whom the approach did and did not work well; and what facilitators and barriers exist to successfully applying eLearning. The evaluations were designed to provide the Senegal and Uganda ministries of health with data to help inform decisions about scaling up provider training while also contributing to broader learnings for innovative training approaches in the context of new contraceptive introduction.

This article describes findings from a multicountry evaluation of an eLearning program for the introduction of DMPA-SC.

## ELEARNING COURSE DESCRIPTION

In response to the growing interest in this new product and the pressing need for distanced and affordable training approaches, the PATH-led Injectables Access Collaborative (https://fpoptions.org/topics/ac-lan/), in collaboration with an instructional design firm, developed the DMPA-SC online training course family planning providers learning to offer DMPA-SC, including through SI. The Access Collaborative, formerly known as the DMPA-SC Access Collaborative, provides technical assistance to countries that are introducing DMPA-SC for SI. Based on PATH’s original classroom training curriculum, the eLearning course is available in French and English and includes 10 lessons: (1) what DMPA is, (2) what Uniject is, (3) the difference between DMPA-intramuscular (IM) and DMPA-SC, (4) how to screen clients who wish to use DMPA-SC, (5) how to counsel clients about DMPA in the context of informed choice, (6) how to safely store and handle DMPA-SC, (7) how to give a DMPA-SC injection, (8) how to calculate the reinjection date, (9) how to conduct client follow-up visits, and (10) how to counsel clients on DMPA-SC SI. The course was built using Articulate 360 software and is available on the Population Services International’s Kassai platform (https://www.kassai.org/). During the evaluation, trainees registered on the online platform that tracked participation and completion of course modules. PATH developed and distributed a job aid with directions to access the course for participants in each country.

Most modules take approximately 15–20 minutes to complete, except for the SI module, which takes approximately 1 hour. Thus, the entire 10-module course takes approximately 4–5 hours. The course includes interactive exercises and short quizzes at the end of each module to assess comprehension. A pre-/post-test and final quiz track mastery of the material. An 80% score on a lesson quiz advances the learner to the next lesson. Scoring at least 80% on all quizzes, as well as the final quiz, earns a certificate of completion. The training can be taken on any device with Internet connectivity, including a smartphone connected to a cell network or Wi-Fi. Additionally, because every user has a unique username and profile, completion rates can be tracked by facility, district, organization, and type of provider, allowing the Ministry of Health (MOH) to track provider training progress and identify facilities with training gaps.

### Uganda Context

Since 2003, Uganda has been exploring how to increase the availability of family planning services and meet its Family Planning 2030 goals. Central to this effort has been delivering DMPA-SC through diverse delivery channels including directly to communities through community health workers (CHWs), called village health teams, which began in 2014. With policy approval for SI in 2020, training ramped up in the public sector. More trained private- and public-sector providers are required to advance from introduction to national scale. To address these training needs, PATH implemented the eLearning program in 2019 in 4 districts in Uganda: Luwero, Mayuge, Nakasongola, and Kayunga, enabling private and public providers to receive training in both how to counsel women for DMPA-SC and initiate SI. Providers who had not previously been trained in DMPA-SC administration, possessed or had access to a smartphone or computer, and were literate in English were eligible to register for the course. To recruit potential trainees, PATH worked with the district health teams to identify providers in the public sector who had not benefited from previous trainings on DMPA-SC. The district health teams also helped to identify pharmacies, drug shops, and private clinics to whom PATH reached out to issue invitations to participate in the eLearning course.

### Senegal Context

In Senegal, a progressive rollout of DMPA-SC was launched at all levels of Senegal’s public-sector health system in 2014, and the MOH approved SI in 2018. While training of facility-based public-sector providers in DMPA-SC administration is complete, as of 2019, many providers lacked training in how to counsel women for SI. A gap in funding for 5 regions (Kaffrine, Kaolack, Fatick, Louga, and Ziguinchor) impeded the MOH from achieving national scale-up of SI, prompting PATH to offer technical assistance to implement eLearning module 10 (focused on SI) in these regions. Providers who had not previously been trained in how to counsel women for SI, possessed or had access to a smartphone or computer, and were literate in French were eligible to register for the course. To recruit training participants, PATH organized orientation meetings in each region to introduce eLearning to provincial and district leadership, who, in turn, notified public sector providers of the opportunity to learn SI via eLearning.

In both countries, selected geographies for eLearning reflected areas that were geographically accessible, where Internet and cell network connectivity was relatively good, and where many providers from public and private sectors had not yet been trained in SI. District health officials identified providers who were eligible for training. Participants received mobile phone data stipends for Internet costs and were given 3–5 weeks to take the course. Providers were assisted with registration procedures for the course via a job aid in written and video format. Upon completion, district supervision teams provided post-training supportive supervision to providers to assess and strengthen their skills and comprehension, and complete evaluation activities. Supportive supervision served to address questions, clarify misperceptions, and confirm injection and/or SI counseling proficiency; upon satisfactory confirmation of skills, providers received certificates.

### Training Approach

Beyond these parameters, the eLearning approach differed somewhat between the 2 countries. In Uganda, both public- and private-sector providers were invited to take the full course, with 10 modules covering both DMPA-SC administration as well as SI counseling. Providers who were identified as having not previously been trained in DMPA-SC and SI were invited to participate, and only those who completed the course were invited to the post-training supportive supervision. Table 1 outlines the principal similarities and differences between the eLearning course approach in each country.

**TABLE 1.** Differences in eLearning Course Approach by Country

	**Senegal**	**Uganda**
Provider profile	Public-sector clinic providers	Public and private clinics, drug shops, pharmacies
Provider experience	Experienced with subcutaneous DMPA	Subcutaneous DMPA naïve
	Not previously trained to counsel women for self-injection	
Support provided	Data plans, registration job aids, and focal point-persons for technical assistance	
Provider requirements	Smartphone or computer, able to read French or English	
Post-training follow-up	Post-training supportive supervision	
Alternative to eLearning	On-the-job training for those who did not take the eLearning	No alternative offered

In Senegal, public sector providers were invited to take a single module focused on SI (module 10). Providers who did not take the eLearning course were offered in-person on-the-job training. This approach made it possible to compare evaluation outcomes for eLearning trainees relative to in-person trainees. Both groups received post-training supportive supervision 3–5 weeks after training.

A total of 402 providers in Senegal and 570 providers in Uganda enrolled in the eLearning course (Table 2). Completion rates differed considerably, with 84% completing the single module on SI in Senegal, while 48% completed the full 10 modules on DMPA-SC, including the SI module in Uganda. Challenges around course completion were explored in the evaluation.

**TABLE 2.** Course Offering, Enrollment, and Completion by Country

	**eLearning course**	**Enrolled, No.**	**Completed, No. (%)**
Senegal	Self-injection module	402	339 (84.3)
Uganda	Full course	570	271 (47.6)

A total of 402 providers in Senegal and 570 providers in Uganda enrolled in the eLearning course.

## METHODS

### Study Design

Using a mixed-methods approach, the evaluation assessed the extent to which online training is effective, feasible, acceptable, and cost-efficient for teaching providers how to administer DMPA-SC (Uganda) and how to counsel clients for SI (Uganda and Senegal). The study team collected data to understand training effectiveness (measured by injection proficiency in Uganda, ability to train clients to self-inject in Senegal, and overall knowledge acquisition in both countries), feasibility and practical constraints (barriers and challenges to successful completion of the course), acceptability (measured in terms of provider training approach preferences and satisfaction), and in-country program implementation costs. Quantitative data were collected through structured surveys, post-tests, a final quiz, and an observation checklist and qualitative data were collected through interviews.

The evaluation also collected information about the cost of implementing eLearning to train providers in both countries and in-person training in Senegal. Costs were categorized into 3 primary activities: (1) orientation: national and regional workshops to introduce the program and build buy-in; (2) training: preparation, registration, support to learners, and other associated costs (e.g., data bundles for learners); and (3) post-training supervision: follow-up visits for post-training coaching. Only costs specific to implementation in Senegal and Uganda were included, and the costs of developing the eLearning content were not captured in the analysis.

### Participant Recruitment and Sample Size

From the 570 total enrollees in Uganda, 261 were randomly selected for the evaluation, including 208 selected during the post-training supportive supervision from among those who completed the eLearning course and a smaller sample (53) selected from among those who registered but did not complete the course. The sample of participants who did not complete the eLearning course were contacted via phone for an interview.

In Senegal, 397 participants were randomly selected for the evaluation at the supportive supervision visit, with roughly even samples of participants who completed eLearning (n=196) and those who received in-person training instead (n=201).

### Data Collection Procedures and Analysis

After consenting to the evaluation, participants who completed training took a post-training knowledge assessment (post-test) at the start of the supportive supervision visit to measure knowledge retention. The 15-item post-test was multiple choice and included questions on DMPA-SC contraindications, side effects, critical steps in administering DMPA-SC, counseling clients for SI, and the DMPA-SC reinjection window. The same post-test was administered in both countries.

In Uganda, those who completed eLearning were asked to demonstrate their ability to give a DMPA-SC injection correctly, without any additional guidance or coaching, during the supportive supervision visit. An observation checklist was used to assess their proficiency. To qualify as competent, providers needed to demonstrate correctly (on a model) the 4 critical steps: (1) shake the device to mix the product, (2) activate the device to break the seal between the reservoir and needle, (3) tent the skin to ensure a subcutaneous injection, and (4) squeeze the reservoir slowly to inject the drug.

In Senegal, all providers participated in an observational assessment where they performed a mock SI counseling session as a role-play during supervision. For the observation of a mock SI counseling session, there are 11 steps in the assessment, 5 of which are “critical”: (1) demonstrate injection technique while client follows along; (2) supervise, support, and correct while client tries SI using job aid or instructional video; (3) train client to calculate reinjection date; (4) counsel client on proper storage; and (5) provide client with necessary materials. Competent providers scored “satisfactory” on all 5 critical steps. Because of an unintended difference between groups in how the observational assessment was administered, in-person learners had the opportunity to practice before supervision, whereas the eLearners did not. Because any training group difference may be due to the timing of proficiency measurement (before or subsequent to practice), only the results for eLearners are included in the results. 

Participants in both countries were also interviewed in person via a structured questionnaire about their experience with the eLearning course (and in-person training in Senegal). The survey for eLearners captured information on how participants engaged with the course, identified challenges experienced while taking the training, as well as any costs incurred, and measured overall acceptability, satisfaction, and training preferences. A summary of concepts measured and tools employed in each country is shown in Table 3.

**TABLE 3.** Summary of Concepts Measured and Corresponding Instruments in Each Country

**Concept Measured**	**Tool Employed**	**Senegal**	**Uganda**
Knowledge acquisition	Post-test	X	X
Competency	Observation checklists		
Injection proficiency	Injection administration		X
Self-injection counseling	Counseling session	X	
Individual characteristics, including prior subcutaneous DMPA training and experience	Structured survey	X	X
Approach to eLearning (modalities for taking the course)	Structured survey	X	X
Acceptability / training preferences	Structured survey	X	X
Provider perspectives	In-depth interviews	X	X
Cost of training	Structured survey (trainees)	X	X
	Costing tool (program records)	X	X

Finally, program costs were calculated using a costing tool that captured programmatic costs, such as staff time, transportation and per diem, and data support for trainees.

Data collection instruments were programmed using Open Data Kit software, and data were entered on mobile phones simultaneously (survey interview) or collected on paper (post-test and observations) and subsequently entered in Open Data Kit. Data quality was assessed biweekly to identify and correct common errors and examined rates of course completion, mastery of the material, injection proficiency, acceptability by cadre and sector, and the costs of offering and supporting eLearning. Qualitative interviews were conducted in the preferred language of the participant, audio-recorded, translated, and transcribed verbatim.

### Data Analysis

The structured survey data were analyzed using STATA version 14 to assess levels of mastery of course material. Differences between the facility and community-based providers (Uganda) and between providers trained via digital and in-person approaches (Senegal) were assessed using chi-square and Student’s t-tests, with conventional significance levels of 95% by 2-tailed tests. Additionally, the cost of providing and supporting online training was calculated in total and by activity. The in-depth interview data were analyzed manually based on an inductive coding system developed by the analysis team. Multiple team members were involved in the analysis, and consensus was reached around key themes and their applications. The qualitative analysis identified the main reasons for not completing the training or mastering its content and the difficulties faced by participants.

### Ethical Approval

All participants provided voluntary, written, informed consent to participate in the evaluation. In Uganda, the Mulago Hospital Research and Ethics Committee and the Uganda National Council for Science and Technology approved this research study. In Senegal, the Ministry of Health and Social Action’s National Ethics Committee for Health Research approved the study.

## RESULTS

### Participant Background

With respect to participant characteristics, the typical Ugandan participant was a female nurse or midwife, average age of 30 years, who had been working in family planning for 5 years. Over half (55%) of eLearners were from the private sector, including 40% from private clinics and 15% from pharmacies/drug shops. CHWs represented 13%. In Senegal, the typical eLearning participant was a female midwife in the public sector, average age of 35 years, who had been working in family planning for 7 years. In both countries, the majority (85%–86%) had no prior experience with online training.

By design, eLearners in each country had different levels of experience with DMPA-SC (Table 4). In Senegal, the training targeted providers familiar with DMPA-SC, and 92% were very experienced with DMPA-SC administration. In Uganda, eLearning was intended to benefit providers not yet trained in DMPA-SC administration. Though just 11% had received formal training in DMPA-SC, 28% were nonetheless experienced with DMPA-SC administration. In both countries, only about one-third of providers had heard of SI.

**TABLE 4.** Experience of eLearners With Subcutaneous DMPA and Self-Injection by Country

	**Senegal, No. (%)** **(N=196)**	**Uganda, No. (%) (N=208)**
Heard of subcutaneous DMPA	188 (95.9)	177 (85.1)
Heard of subcutaneous DMPA self-injection	66 (33.7)	74 (35.6)
Number of subcutaneous DMPA injections given prior		
Never	2 (1.0)	106 (51.0)
1–2	5 (2.6)	17 (8.2)
3–10	9 (4.6)	27 (13.0)
More than 10	180 (91.8)	58 (27.9)
Previous training in subcutaneous DMPA	144 (73.5)	66 (31.7)
Previous subcutaneous DMPA training type		
None	52 (26.5)	142 (68.3)
Informal training by a colleague	20 (10.2)	41 (19.7)
Formal training, including in person	124 (63.3)	23 (11.1)
Other	1 (0.5)	2 (1.0)

### Course Effectiveness

Participants scored relatively well on the 15-item test of DMPA-SC knowledge, with mean scores of 12–14, depending on country and training modality. Senegal providers scored lower than providers in Uganda (Table 5), reflecting the abbreviated nature of their training (SI module only). Providers were most challenged by a question about the acceptable timing for reinjection; just 38% in both countries understood that a pregnancy test was not needed if a client returned for reinjection up to 4 weeks late (data not shown). Fifteen percent of Ugandan providers and 18% of Senegalese providers erroneously believed that women who had not been to school were unable to self-inject (data not shown). There was no statistically significant difference in post-test scores between Senegal eLearning trainees vs. in-person trainees.

**TABLE 5.** Participant Scores on DMPA Knowledge Test

	**Senegal eLearners (N=196)**	**Senegal In Person Learners (N=201)**	**Uganda eLearners** **(N=208)**
Mean score, % (range)	12.4 (8–15)	12.1 (5.7–15)	14.0 (8.5–15)
Questions correct, no. (%)			
80%	126 (64.3)	110 (54.7)	184 (88.6)
75%	157 (80.1)	147 (73.1)	196 (94.2)
70%	180 (91.8)	170 (84.6)	200 (96.2)

Participants scored relatively well on the 15-item test of DMPA-SC knowledge, with mean scores of 12–14, depending on country and modality.

### Senegal Self-Injection Observational Assessment

As shown in Table 6, just 17% of eLearners completed all 5 critical steps correctly. For each of these 5 steps, between 46% and 84% of the eLearners could complete the step satisfactorily. In step 5, providers struggled with proper technique while activating the device or demonstrated poor injection technique during the observation. Scores were lowest for step 6; the most common error was not having the client practice injecting on a model. eLearners commonly skipped step 7, not describing the reinjection window, and some providers did not teach the client how to calculate reinjection dates. Providers commonly skipped counseling the client on how to store the product at home. Providers were more successful with counseling clients on disposal practices.

**TABLE 6.** eLearners’ Proficiency in Self-Injection Client Counseling, Senegal

**Steps (critical steps in bold)**	**No. (%) (N=196)**
1. Counsel the client on family planning options, provide desired method.	163 (83.2)
2. For clients interested in self-injection, determine eligibility for subcutaneous DMPA.	134 (68.4)
3. Counsel the client on side effects.	152 (77.6)
4. Prepare supplies and training aids.	114 (58.2)
**5. Demonstrate injection technique while client follows along with job aid; emphasize the 4 critical injection steps.**	**101 (51.5)**
**6. Supervise, support and correct while client tries self-injection using job aid or video. Confirm client completed 4 critical steps.**	**90 (45.9)**
**7. Train client to calculate reinjection dates using calendar and job aid.**	**111 (56.6)**
8. Counsel the client on proper storage.	124 (63)
**9. Counsel client on proper disposal.**	**165 (84)**
10. Provide client with necessary materials (e.g., job aid).	107 (54.6)
**11. Explain to client when to follow up.**	**129 (65.8)**
Completed all 5 critical steps.	33 (16.8)

During an observational assesssment of SI counseling steps, just 17% of eLearners in Senegal completed all 5 critical steps correctly.

### Uganda Injection Proficiency Assessment

As shown in Table 7, 71% of participants who completed the eLearning demonstrated injection proficiency (administered the injection consistent with the 4 critical steps). When restricting the analysis to those who had never previously administered an injection, 69% demonstrated proficiency. At least 90% of providers performed injection steps 3, 4, and 7 correctly. Among the 4 critical steps, providers were most challenged by step 6, pinching the skin to create a tent (for subcutaneous injection), with 81% demonstrating competence.

**TABLE 7.** eLearners’ Proficiency in Subcutaneous DMPA Administration, Uganda

**Critical Steps**	**No. (%) (N=210)**
3. Mix the liquid by shaking the device for 30 seconds.	189 (90.0)
4. Push the needle cap and port together to activate the Uniject.	198 (94.3)
5. Pinch the skin to form a tent and insert needle.	174 (82.9)
7. Press the reservoir slowly to inject for 5–7 seconds.	195 (92.9)
Completed all critical steps.	150 (71.4)

**FIGURE 1:**
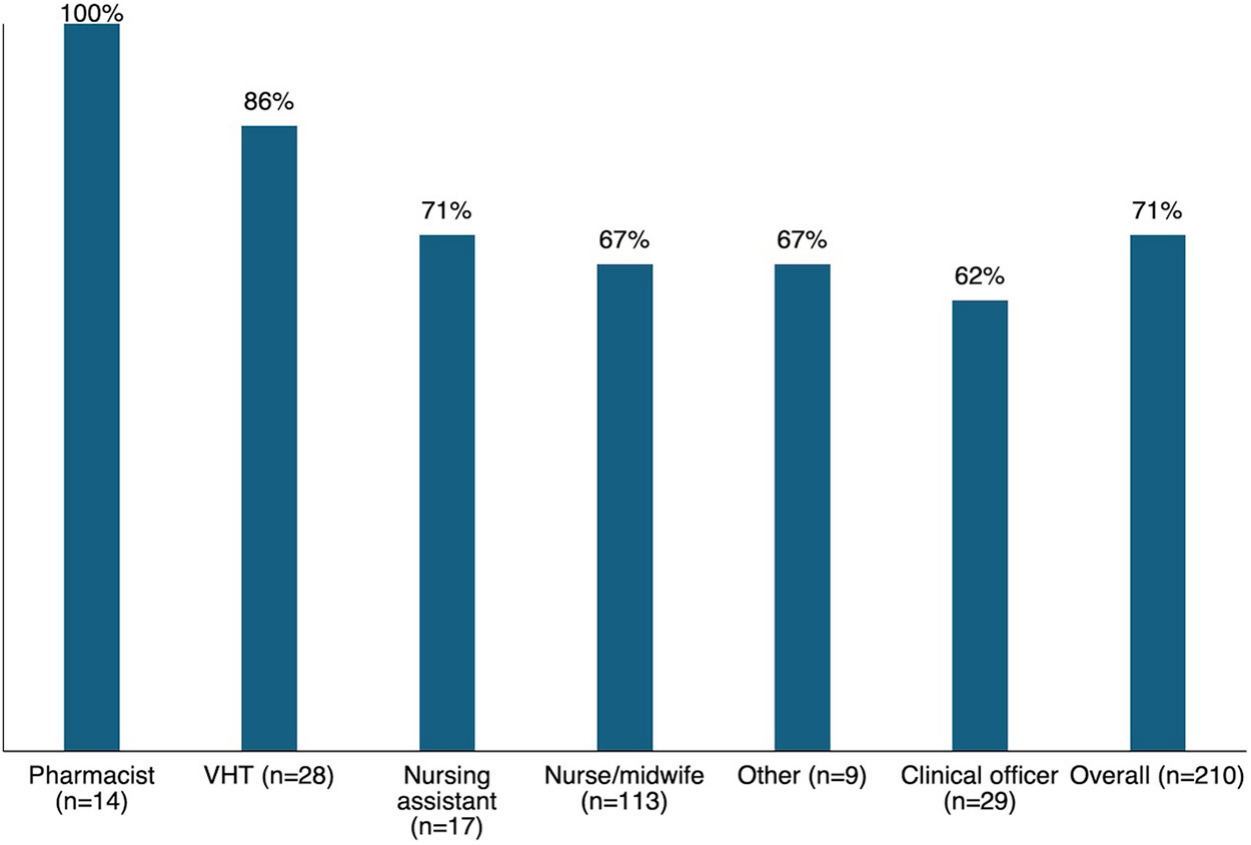
Injection Proficiency After eLearning by Provider Cadre,^a^ Uganda Abbreviation: VHT, village health team. ^a^Other category includes additional cadres such as medical records assistants, health information assistants, and peer mothers.

Differences in injection proficiency by provider cadre are shown in Figure 1. Though few in number, 100% of pharmacists and lab technicians (n=14) were statistically significantly more likely to demonstrate competence relative to all other cadres of health workers. About 67% of nurses and midwives demonstrated competence, as did 86% of village health teams. Overall, there was no difference in proficiency between public and private-sector providers.

### Approach to eLearning

In both countries, most participants took the course during their free time before or after work (Table 8). In Senegal, providers were unable to use time during their work hours to take the course (just 11% took the course at work). In Uganda, 42% took the course while at work, primarily in the afternoon when there were fewer clients.

**TABLE 8.** Approach to eLearning During Work Hours or Non-Work Hours

	**Senegal, No. (%) (N=196)**	**Uganda, No. (%) (N=208)**
Took course during non-work hours	174 (88.8)	150 (57.7)
Took course during work hours	22 (11.2)	110 (42.3)
Able to adjust workload	67 (34.2)	67 (25.8)

In Senegal, 24% of participants elected to take the course in a group, and the majority (57%) reported that they preferred working with others to discuss, motivate, and help one another (not shown). Providers who took the course alone felt that it allowed them to concentrate better and was less distracting.

### Challenges With eLearning

Over half of the participants in Senegal reported they experienced no challenges with the eLearning course; however, about 45% of eLearners in Senegal and 77% in Uganda faced challenges (Table 9). Internet connectivity was the most common issue faced by 50% of participants in Uganda and 29% in Senegal, and website navigation was problematic for 18% of participants in Senegal.

**TABLE 9.** Challenges Encountered by eLearning Participants

	**Senegal, No. (%) N=196**	**Uganda, No. (%) N=208**
Nothing challenging	107 (54.6)	47 (22.6)
Connectivity problems	57 (28.9)	104 (50.0)
Website/navigation	36 (18.3)	12 (12.0)
Not enough data (data plan insufficient)	6 (3.0)	15 (7.2)
Content unclear/difficult to understand	6 (3.0)	13 (6.3)
Not enough time	5 (2.5)	35 (16.8)
No device/had to borrow a device	5 (2.5)	32 (15.4)
Language difficulties	1 (0.5)	6 (2.9)

### Provider Preparedness to Counsel Clients on Self-Injection

After training but before supervision, when asked how prepared they felt to train clients to begin SI, providers in Senegal who received in-person training reported statistically significantly higher preparedness than did eLearners (72% felt “very prepared” as compared with just 56% of eLearners) (Table 10). This pronounced difference in perceived readiness disappeared in both groups after the post-training supportive supervision, at which time 99% of both groups reported feeling very prepared. We see a similar statistically significant pattern in Uganda, where the percentage that felt “very comfortable” administering a DMPA-SC injection increased from 45% immediately post-training to 93% after supportive supervision (Table 11).

**TABLE 10.** Provider Preparedness to Offer Self-Injection and Subcutaneous DMPA, Senegal

	**%**		
	**Very Prepared**	**Somewhat Prepared**	**A Little or Very Unprepared**
Before supervision			
eLearners (N=196)	56.2^a^	37.6	6.2
In-person learners (N=201)	72.1	24.9	3.0
After supervision			
eLearners (N=196)	98.5	1.6	0.0
In-person learners (N=201)	98.5	0.0	1.5
Uganda			

^a^Significant difference from in-person learners at the P<.05 level.

**TABLE 11.** Uganda eLearners Preparedness to Offer Subcutaneous DMPA

	**%**			
**Injection inexperienced providers only, n=119**	**Very Comfortable**	**Somewhat Comfortable**	**Somewhat Uncomfortable**	**Very Uncomfortable**
Before supervision	45.4^a^	35.3	11.8	7.6
After supervision	93.3	6.7	0	0

^a^Significant difference in percentage very comfortable before and after supervision.

### Provider Training Preferences

**FIGURE 2:**
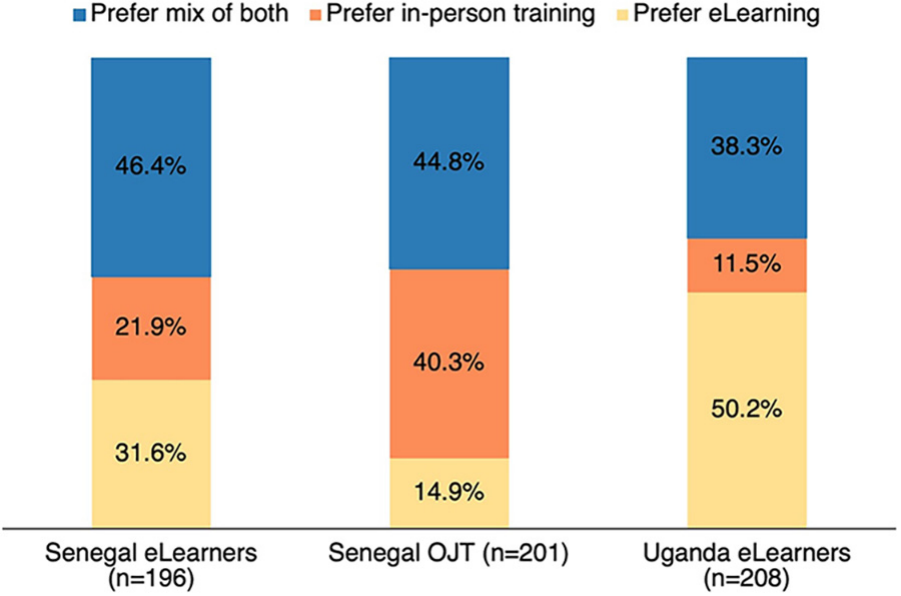
Provider Training Preferences Abbreviation: OJT, on-the-job.

In terms of training preferences, 32% of Senegalese and 50% of Ugandan eLearners preferred eLearning to in-person training (Figure 2). A substantial share of eLearners in both countries prefer a blend of the 2 approaches. In contrast, just 15% of those who received in-person training reported that they would prefer eLearning. In Uganda, CHWs were more likely to favor a mix of eLearning and in-person approaches; male and female providers were not statistically different in their training preferences (not shown).

Over 30% of participants in Senegal and 50% in Uganda preferred eLearning over in-person training.

### Provider Perspectives on eLearning: Training Preferences, Advantages, and Drawbacks

In qualitative interviews, respondents elaborated on the reasons for their training preferences, highlighting the flexibility and convenience of eLearning as well as opportunities for interaction, practice, and questioning offered by in-person training.

*The 2 approaches are complementary. Regarding the online course, you can arrange your time to follow the training. With traditional training the person [trainer] is in front of you which allows you to understand the lesson or the module more quickly and, in my opinion, you have more focused concentration. You are not obliged to respond to clients or children who come to the clinic. We have to concentrate to understand what the person is telling us. The best benefit to us is to associate online course with traditional training.* —Midwife, eLearning participant, Senegal

*I would pick neither, because I like both. Like in medical [school], we normally have practical part. So, I want to learn theory, then I do eLearning … then we arrange and do the practicals [in person].* —Clinical officer, Uganda

Providers shared perceived advantages of eLearning, including that it saves time and eliminates the need to travel or leave the worksite, which is of particular benefit for providers who live far from central training sites. They also reported that it allows for better concentration than in a group setting, offers flexible timing, encourages providers to “keep up with technology,” and covers a lot of material in a short time.

Providers shared perceived advantages of eLearning, including that it saves time and eliminates the need to travel or leave the worksite.

*Online training allows the provider to minimize leaving their worksite so as to better satisfy sick patients to be trained in record time and at lower cost.* —Nurse, in-person training participant, Senegal

*I would prefer eLearning because I don’t think I would get free time to attend those [in-person training]. I do it in my free time, like now I am at duty. I get a patient, I attend to a patient, then I go back to where I stopped.* —Midwife, private clinic, Uganda

*[eLearning] allows us to save time. We are still at the health post and we can continue ensuring our consultations as well as the course. We won’t abandon the clinic to spend a whole day in a hot classroom. In any case, we are more comfortable.* —Nurse, eLearning participant, Senegal

*Online training is a good approach particularly at this moment with the coronavirus when we need to avoid group gatherings. Even if some areas have poor Internet, I’m sure that in a few years’ time more and more trainings will be online. —*Midwife, eLearning participant, Senegal

Drawbacks cited by providers include that the platform needed to be easier to access, as enrollment required a multistep process. The original enrollment process on the eLearning platform was overly complicated, a challenge that was addressed post-evaluation by switching to a new host site. Providers also reported that eLearning only worked in areas where Internet connectivity was strong, many providers lacked computers and smartphones or had trouble navigating the course, and training was often interrupted by work requirements.

*Personally, I don’t think it’s possible for the time being because all conditions are not in place. First, providers don’t have computers, many don’t have access to Internet, and there are providers in the far zones of Senegal where Internet connectivity fails. Sometimes you have to get up at 1 or 2 o’clock in the morning to get a connection.* —Midwife, eLearning participant, Senegal

*I would get stuck somewhere. Maybe the problem is the phone, I don’t know. I get stuck somewhere and they don’t show me where to continue. Maybe I don’t know which other step to go to, then I would get some advice, maybe ask my friend or my other friend there, or you, when you come then we consult you then we go on.* —Private midwife, Uganda

### Comparative Analysis of Training Costs

As shown in Table 12, eLearning had a substantially lower per person cost in Uganda (US$39) relative to Senegal (US$66). Note that costs are calculated inclusive of post-training supportive supervision. The main cost driver in Senegal was the orientation, which included transportation and travel costs for meetings with district and regional health authorities. In Uganda, costs were more evenly distributed across activities, though supporting eLearners (with registration and tech support) was the most expensive cost category at US$16 per participant. Within broad cost categories, primary cost drivers were per diem during orientation (US$16/person), transportation to conduct post-training supervision (US$11/person), and airtime/data for training (US$9/person). Travel-associated costs (transportation, per diem, and lodging) are primary cost drivers, accounting for 63% of all costs. Staff time was a secondary driver at 23% of total costs. Uganda’s overall lower cost can be attributed to lower transportation/travel costs for orientation sessions and post-training supervision. District and regional orientation sessions for local health authorities are cost drivers, and reaching more providers through e-learning could lower per-person costs. Similarly, staff time does not increase proportionally with the number of participating trainees.

eLearning had a substantially lower per person cost in Uganda compared to Senegal.

**TABLE 12.** Training Cost Averages in Senegal and Uganda^a^

	**Senegal**		**Uganda**
	**eLearning**	**In Person**	**eLearning**
Total cost per person^b^	$66	$134	$39
Orientation to eLearning, cost (percentage of total)	$26 (39.9)	$55 (40.7)	$12 (29.8)
Supporting participation, cost (percentage of total)	$18 (27.4)	$28 (20.9)	$16 (39.7)
Post-training supervision, cost (percentage of total)	$21 (31.8)	$52 (38.4)	$12 (29.4)

^a^All costs are in U.S. dollars and rounded to the nearest dollar.

^b^Cost includes orientation of regional and district personnel.

In Senegal, costs of eLearning were compared to costs of in-person learning. eLearning had a substantially lower per person cost than in-person training due to the higher number of participants (337 vs. 229) and lower transportation/travel cost (data not shown).

### Future Implementation Costs

The team undertook a supplemental analysis to estimate cost savings that could be incurred if eLearning were scaled up in Senegal. That analysis reflected that the estimated base cost per additional region launched would be US$3069 plus US$43.92 per provider trained. Greater efficiencies in regional orientations could further reduce the total cost of scaling to new regions. These costs represent estimates for implementation during non-COVID times (e.g., each region would have a single, 1-day orientation) given that due to the COVID-19 pandemic, certain activities were prolonged and/or incurred increased costs (e.g., multiple orientations were held for each region to allow for social distancing). For both countries, future costs could be lower if efficiencies were gained through scale and implementation lessons were considered, including streamlining the registration and monitoring via an improved eLearning platform that would reduce the need for learner support.

## DISCUSSION

### Factors Necessary for Successful eLearning

eLearning has been used in a variety of health contexts in LMICs in the sexual and reproductive health field, from postgraduate training in obstetrics-gynecology in Argentina to HIV index testing in Malawi and training midwifery students in Rwanda.^15^^–^^17^ Generally, the literature finds that eLearning can increase educational opportunities, efficiency, and effectiveness. However, a number of factors still present particular challenges for eLearning in LMICs, including institutional characteristics (limits of infrastructure and human resources), information technology support (quality and speed of connectivity), and supportive learning environment (limited access to mobile technology, level of interaction with instructors and other learners, support provided to eLearners for data plans).^1^^,^^3^^,^^7^^,^^8^^,^^14^^–^^17^Additional factors—found in this study and others—that impact the success of eLearning programs include instructor and learner characteristics (such as motivation to acquire new skills, digital literacy, learning needs, attitudes toward eLearning, and ability to adjust workload to complete the course) and the program design (ease of registering for the course or the availability of a registration job aid, synchronous or asynchronous options, and content and length. The results of this study—and in particular, the large increase in providers’ sense of preparedness following supportive supervision—support the findings in the literature that suggest that digital training for health workers is optimal when coupled with supportive supervision that includes a practicum or complemented by an abbreviated in-person training.^1^^,^^5^^,^^10^^,^^18^ In whatever form training takes, supportive supervision to solidify learning should be part and parcel of the training program.

This evaluation demonstrates that deploying a strong eLearning program at the district level requires addressing these technical and environmental challenges to ensure ease of access and program effectiveness. One critical component is to initiate coordination among stakeholders at the MOH and regional or district health offices and facility leads early in the planning process. Particularly at the facility level, eLearning champions who support health workers can be catalysts for success, helping providers overcome common challenges by providing enrollment guidance, technical orientation, troubleshooting, and reminder messages. For recognition of training completion, the printing, signing, and official presentation of certificates adds value to the training and may contribute to greater participation, as suggested by the reaction of providers who received them. Additionally, gathering and aggregating feedback from eLearners will allow stakeholders to iterate on their eLearning program and improve its quality.

Can eLearning replace in-person training? We found that eLearning can play an important role, potentially reducing training costs but must be coupled with the appropriate supports. Given persistent limits of connectivity, access to technology, and digital literacy in many LMIC settings, not all providers will be well-positioned to succeed with eLearning, suggesting that in-person training will continue to be important. Post-training supervision for eLearning trainees is crucial, as demonstrated by the increase in provider preparedness pre- and post-supportive supervision. This finding aligns with the World Health Organization’s recommendation and the findings of other evaluations for digital training. eLearning should complement, rather than replace, traditional in-service training for health systems strengthening.^5^ Supportive supervision was instrumental in helping health workers apply their course knowledge to hands-on practice offering DMPA-SC and SI counseling, as evidenced by significant increases in readiness to offer services after supervision. In Senegal, lower proficiency scores on the more practical critical SI client counseling steps 5–7 and the proportion of eLearners in both countries who felt very prepared to offer DMPA-SC and SI after finishing the course and receiving supervision nearly doubled, underscoring the importance of a hybrid approach as post-training supervision solidifies learning and ensure readiness to offer the service. Future evaluation is merited to explore the degree to which, when blended with in-person training and supportive supervision, eLearning could enable programs to reduce the number or length of traditional training and ensure continuity of care at facilities while permitting providers to learn clinical and technical skills.^17^

We found that eLearning can play an important role, potentially reducing training costs, but must be coupled with the appropriate supports.

### Limitations

The course was hosted on a site that lacked the capacity to collect the full registration information required to adequately track participants. In Senegal, this necessitated supplemental registration through PATH and through the MOH’s continuing education site with links embedded to the host site. Additionally, the content was not available for offline viewing, thus presenting limitations for eLearners with no or irregular access to a stable Internet connection. The complicated registration process likely contributed to lower enrollment. After the evaluation, the course was migrated to a different hosting site with an easier-to-access system.

The number of CHWs who completed the course in Uganda (28) may not be typical or representative and doesn’t permit us to draw conclusions as to why this group scored higher than nurses and midwives on the assessment of injection proficiency.

As previously noted, the in-person training group in Senegal was given the opportunity to practice counseling for SI before their evaluation, which confounded the comparison of proficiency between eLearners and in-person learners. Consequently, proficiency results for in-person learners were excluded from the article.

## CONCLUSION

This evaluation contributes to burgeoning literature on digital health interventions in LMICs and provides insights into best practices for using eLearning training approaches to maximize resources in training health workers. The results underscore the importance of supervision and the use of eLearning to complement, not replace, traditional health education and training approaches. eLearning is not a universal solution, and like many digital health interventions, it may be less feasible for health workers in settings with poor network coverage and access to technology or for those who lack digital literacy.^1^^,^^3^^,^^6^^,^^7^^,^^12^^,^^18^ However, it also presents advantages, such as decreasing costs and reducing the need for or duration of in-person trainings when geographical distance or health events like COVID-19 present logistical challenges, that could prompt MOHs to adopt it as an approach. Future research is merited to assess the long-term retention of knowledge and skills gained through eLearning approaches.

Digital interventions will be most successful when investments are made to expand beyond pilots and incorporate solutions into health care systems at scale, which requires government and donor support, planning, and iterative improvement.^19^ When implemented at a larger scale, the costs of developing and deploying eLearning are amortized. Successful implementation of digital health interventions in LMICs requires end-user input, stakeholder engagement and motivation, simple operability, alignment with a broader policy environment, and appropriate digital infrastructure.^20^

## Notes

**Peer Reviewed**

**First Published Online:** September 13, 2024.
